# Anthropogenic noise variation in Indian cities due to the COVID-19 lockdown during March-to-May 2020a)

**DOI:** 10.1121/10.0006966

**Published:** 2021-11-01

**Authors:** A. Mimani, R. Singh

**Affiliations:** Department of Mechanical Engineering, Indian Institute of Technology Kanpur, Kanpur 208 016, Uttar Pradesh, India

## Abstract

This paper analyzes the impact of a nationwide lockdown enforced during March-to-May 2020 to prevent the widespread transmission of COVID-19 on the local anthropogenic noise level variation in Indian cities. To this end, data obtained from the National Ambient Noise Monitoring Network (NANMN) was used to analyze the long-term daily evolution of average day- and night-time levels at selected locations across seven major cities. The results indicate that when the strict lockdown phase 1 was declared, all industrial (I), commercial (C), and residential (R) zones experienced either a gradual or sudden decrease in noise levels while the silence (S) zone was unaffected. Depending on the zone, the weekly trend graphs reached a minimum either during phase 1 or conditionally relaxed phase 2. Across I, C, and R zones, the average maximum day- and night-time reduction with respect to the pre-lockdown period ranged from 4 to 13.8 dB(A) and 4 to 14.1 dB(A), respectively. As anticipated, with a gradual ease in restrictions from phase 2 onwards, the levels climbed back almost linearly, and during unlocks, the daily variation resembled the pre-lockdown trend. Furthermore, the responses to an online COVID-19 noise perception survey supported the NANMN results and suggested that the lockdown was quieter.

## I. INTRODUCTION

Lockdowns or restrictive measures of varying stringency imposed the previous year by the governments of most nations to arrest the spread of the highly infectious coronavirus disease 2019^1^ (COVID-19) that has so far claimed 4.59 × 10^6^ lives worldwide^2^ had a range of adverse socio-economic implications that perhaps may be felt over the next several years. Due to a sudden halt of almost all anthropogenic activities such as industrial and commercial operations, a ban on national and international travel, the closure of schools, universities, entertainment, and leisure places, and a highly restricted outdoor movement resulting in the near-absence of vehicles on otherwise busy roads, the lockdowns produced unintended positive impacts on the environment. This includes a dramatic improvement in air quality in cities^3–5^ as well as a significant reduction in noise pollution levels which altered the urban soundscapes rendering it quieter.

Over the last year or so, there have been several investigations by the acoustic community worldwide on the pandemic-induced environmental noise reduction in different cities and towns.^6–14^ Asensio *et al.*^6^ report a reduction ranging from 4 to 6 dB(A) for the equivalent day, evening, and night indicators and a significant variation in the daily noise patterns, especially during weekends when lockdown was imposed in Madrid (the Spanish capital) during March-to-June 2020. During the same period, Girona, a town in north-east Spain, experienced a drastic reduction in noise levels in the nightlife areas while moderate-to-low changes were observed for commercial and traffic-dense areas, respectively.^7^ Aletta *et al.*^9^ report that London experienced an average noise reduction of 5.4 dB(A) during the spring lockdown in 2020 with respect to (w.r.t.) spring 2019, and there were significant differences in reduction ranging from 1.2 to 10.7 dB(A) across different locations within the city. During the lockdown in Italy, the noise monitoring network in the town of Monza^10^ showed an average reduction between 6 to 10 dB(A) when stringent restrictive measures were in-place (April 2020), while for the capital Rome,^11^ a significant reduction in traffic congestion accounted for the overall noise reduction on the entire road network. In central Stockholm, during the restriction period last year, the levels observed were comparable to those pertaining to the two most popular public holidays in Sweden.^13^ Basu *et al.*^14^ showed that the imposition of the social lockdown in Dublin (Ireland) considerably reduced the traffic volumes resulting in a noticeable reduction in ambient levels. Their analysis was based on studying the daily equivalent day- and night-time level variation at locations spread across the city, and a linear least squares fit indicated a decreasing trend. A few other representative works include a study showing reduced public annoyance due to aircraft noise at residences located near the International Airport at Lima (Peru),^15^ reduction in year-over-year mean weekly underwater noise in deep ocean and inland waters of Canada's Pacific coast due to reduced trade,^16^ reduction in ambient noise at the port of Koper (Slovenia),^17^ and a stronger audible presence of church bells due to reduced human activity in regions of New South Wales, Australia.^18^

Along with analyzing the daily noise data, surveys have also been conducted to quickly gather information regarding people's perception on the changes in soundscape due to the imposition of lockdown and its effect on their well-being and daily routines.^19–22^ The survey results presented in Bartalucci *et al.*^19^ showed that in Italy, perception to traffic noise increased during the lockdown while residents working from home were less annoyed by it. In another study, it was found that people living in multi-unit residential buildings across Canada experienced a somewhat increased annoyance due to the vehicular noise despite a general decrease in ambient levels during the lockdown.^20^ For a quiet residential area in Japan, the survey suggested that the participants perceived the noise levels to be the same during the lockdown and a few weeks after the restrictions were eased.^21^ A survey of over 1000 participants across Turkey revealed that annoyance due to outdoor noise reduced considerably during the lockdown, particularly for busy residential neighborhoods, and it positively correlated with stress and anxiety levels.^22^

An analysis on the effect of COVID-19 induced nationwide lockdown on the environmental noise levels of noisy Indian cities promises to be an interesting study as it presents a unique opportunity to study the Indian urban soundscape in the near-absence of anthropogenic activities for an extended period of time. Yet, such a study has not been reported although some news columns^23^ have been published that mention a significant reduction in noise pollution in major cities. The primary objective of this work is then to analyze the long-term daily evolution of average day- and night-time levels beginning from the pre-lockdown period up to the first few unlock months and to obtain estimates on noise reduction observed at a few representative locations across Indian cities. This paper relies on the environmental noise data continuously recorded by monitoring stations equipped at ten locations each in seven important cities of India whose operations are managed by the Central Pollution Control Board (CPCB), Government of India (GoI), New Delhi.^24,25^ Furthermore, this work also aims to understand, through an online survey, people's perception of the changes in ambient noise levels during the lockdown and correlate it with the noise data.

## II. PHASES OF THE NATIONWIDE LOCKDOWN

The nationwide lockdown imposed by the Ministry of Home Affairs (MHA), GoI across different states and union-territories from 25 March to 31 May 2020 was divided into four phases, beginning from the most stringent part (phase 1) to the most relaxed part (phase 4), which are briefly described below.^26^ Note that the duration from 1 January to 24 March 2020 is referred to as the pre-lockdown period.

• Phase 1: 25 March to 14 April 2020
  During this period, a complete lockdown was enforced and the state governments were asked to strictly adhere to directions from the center. Effectively, all government offices were closed with the exception of police stations, defense and emergency services, and district administration offices. All commercial and private establishments including shopping malls, roadside retail shops, factories, and production units were shut down, and all transport services including airlines, railways, and interstate roadways remained suspended while private vehicles were not allowed to operate. Additionally, hospitality services were completely suspended, educational and research institutions were shut down, places of worship were closed, and a complete ban enforced on socio-cultural and religious gatherings. In essence, outdoor movement was strictly prohibited, thereby confining people to homes resulting in a near-absence of traffic and pedestrians with only essential home deliveries allowed.
• Phase 2: 15 April to 3 May
  Almost all the restrictions imposed during phase 1 continued to be enforced during phase 2, however, GoI assured some conditional relaxations from 20 April onwards subject to the condition that infection was locally contained. The state governments were given greater freedom in formulating the local governance policies to effectively deal with the pandemic. In particular, the relaxations allowed opening-up of agro- and dairy-related businesses, public works, and cargo vehicles began operations. Banks and government centers also reopened but within the constraints of following social distancing and other norms. Furthermore, interstate movement though somewhat restricted was also permitted from this phase. Purely from an acoustical point-of-view, the relaxations signify that the levels start to increase as will be observed from the ensuing results.
• Phase 3: 4 May to 17 May 2020
  For a better management of the pandemic, the entire country was divided into red, orange, and green zones, which were characterized by a big cluster of infection, only a few reported cases, and no confirmed cases in the last 21 days, respectively. The red zones experienced the same restrictions as imposed during phase 1, in the orange zones, only private transport was allowed, while in the green zones much greater relaxations were allowed which included public transportation limited to 50% capacity, and a partial freedom of outdoor movement.
• Phase 4: 18 May to 31 May 2020
  In general, this was the least stringent part where the demarcation of red, orange, and green zones was carried out by the state governments, which also chalked out a future roadmap towards the easing of local restrictions. Note that both phases 3 and 4 witnessed a noticeable traffic volume and commuters which lead to a gradual increase in noise levels.

The unlock (UL) period began on 1 June 2020; the UL 1 duration was from 1 June to 30 June, UL 2 lasted from 1 July to 31 July, and so on. In this work, the total duration from 1 June to 12 August 2020 (data were retrieved up to this date) is collectively referred to as ULs. The reopening during ULs had an economic focus with the lockdown-like restrictions imposed only in the containment zones having a high number of infections. The commercial and industrial establishments were gradually reopened, regular traffic movement and interstate travel as before were permitted, hospitality services resumed, shopping malls and cinemas reopened, and a few higher-educational and research institutions resumed activities as usual while maintaining norms of social distancing.

## III. ENVIRONMENTAL NOISE DATA COLLECTION AND PROCESSING

### A. Central Pollution Control Board (CPCB) noise monitoring stations

The environmental noise data analyzed here was recorded by remote noise monitoring stations or terminals located across seven major Indian cities, namely, New Delhi (the national capital), Lucknow, Kolkata, Hyderabad, Chennai, Bengaluru, and Mumbai, which are the capital of the states Uttar Pradesh, West Bengal (WB), Telangana, Tamil Nadu, Karnataka, and Maharashtra, respectively [refer to part (a) of SuppPub1.jpg^27^]. Each of the seven cities have 10 noise monitoring stations (represented by a tower symbol) which are numbered S1 to S10, and are distributed across important locations or hot-spots as shown in parts (b)–(h) of SuppPub1.jpg.^27^ The exact geographical location, i.e., latitude and longitude along with names of the area where each terminal is located are available on the CPCB website^28^ and may also be found in Refs. 24 and 25. Together, the 70 stations constitute the National Ambient Noise Monitoring Network (NANMN) which is one of the largest of its kind across the globe, and continuously records the ambient noise levels 24 h per day, all 7 days of the week throughout the year. The NANMN is an initiative of the CPCB, New Delhi which is actively involved not only in determining the magnitude of ambient noise levels at important locations but also to take preventive actions to control them. The NANMM program was first established in 2011 and each of the seven metropolitan cities had five noise monitoring stations. In 2014, this network was further strengthened by introducing five additional stations per city. The area in which the terminals are located is categorized in either of the following zones: industrial (I), commercial (C), residential (R), and silence (S) based on the typical daily noise levels. For industrial zones, the maximum allowable limit on the A-weighted equivalent day- and night-time levels denoted $L_{\mathrm{Day}}$ by $L_{\mathrm{Night}},$ respectively, is given by 75 and 70 dB(A), respectively; for commercial zones, it is given by 65 and 55 dB(A), respectively; for residential zones, it is given by 55 and 45 dB(A), respectively; and for silent zones, the limits are 50 and 40 dB(A), respectively. The maximum permissible limits were decided by the CPCB, New Delhi which is the competent authority in India dealing with noise pollution control matters. Out of the 70 noise monitoring locations spread across seven cities, 11 are in I zones, 22 are in C zones, 20 are in R zones, while the remaining 17 are in S zones, and their locations were carefully chosen based on some preliminary short-term noise monitoring surveys conducted by the CPCB and the state pollution control boards. Based on the readings recorded by the terminals, a good estimate of the daily local noise levels can be obtained which in turn can be correlated with the levels at other similar zones, and collectively they shed light on the overall noise pollution for a city over a period of time.

The noise monitoring terminals were manufactured and installed by Geónica Earth Sciences, Spain,^29^ and is a standalone operating remote terminal consisting of a sound level meter traceable to the Indian standards for continuously recording environmental noise. Each terminal consists of a high-quality outdoor microphone compliant with the IEC 61672 Class 1 requirements and is well-protected from wind, rain, birds, and other environmental factors. The microphone is connected to a high-resolution data acquisition (DAQ) system, data logger and an advanced acoustic signal processing unit which computes the A-weighted spectrum and instantaneous (short-duration) sound levels $L_{\text{Aeq}}(t)$ where the sampling time can be user-determined and the options include 0.125, 1, 2, or 10 s, refer to the documentation on the website.^29^ The noise terminals transmits the real-time data using the GPRS mode to the central facility called the National Noise Monitoring Centre (NNMC) located at CPCB Headquarters, Parivesh Bhawan, New Delhi where the graphs of real-time noise levels fed continuously can be plotted. For further details on the NANMN terminals including photographs, the reader is referred to Garg *et al.*^24,25^

### B. Data processing

Based on the instantaneous sound level $L_{\text{Aeq}}(t)$ data pertaining to a short-duration equal to 1 s, i.e., $L_{\text{Aeq},1s}$ received at the NNMC, CPCB headquarters, the hourly equivalent A-weighted sound level $L_{\text{Aeq},1h}$ dB(A) is computed for each hour of a day.^30^ The daily day-time equivalent level $L_{\mathrm{Day}}$ is computed by taking the mean of the hourly levels $L_{\text{Aeq},1h}$ from 6 AM to 10 PM, i.e., over a 16 h duration while the night-time equivalent level $L_{\mathrm{Night}}$ is computed by taking the mean of $L_{\text{Aeq},1h}$ levels from 10 PM to 6 AM (next day), i.e., over an 8 h duration. The noise data is freely available on the CPCB website which was developed exclusively for showing the daily ambient noise levels to the public, and is indeed, the source of data in this work.

## IV. ZONE-WISE ANALYSIS

The analysis of daily noise level evolution graphs is carried out for a few selected noise terminal locations representing different zones.

### A. Industrial and commercial zones

**FIG. 1.**
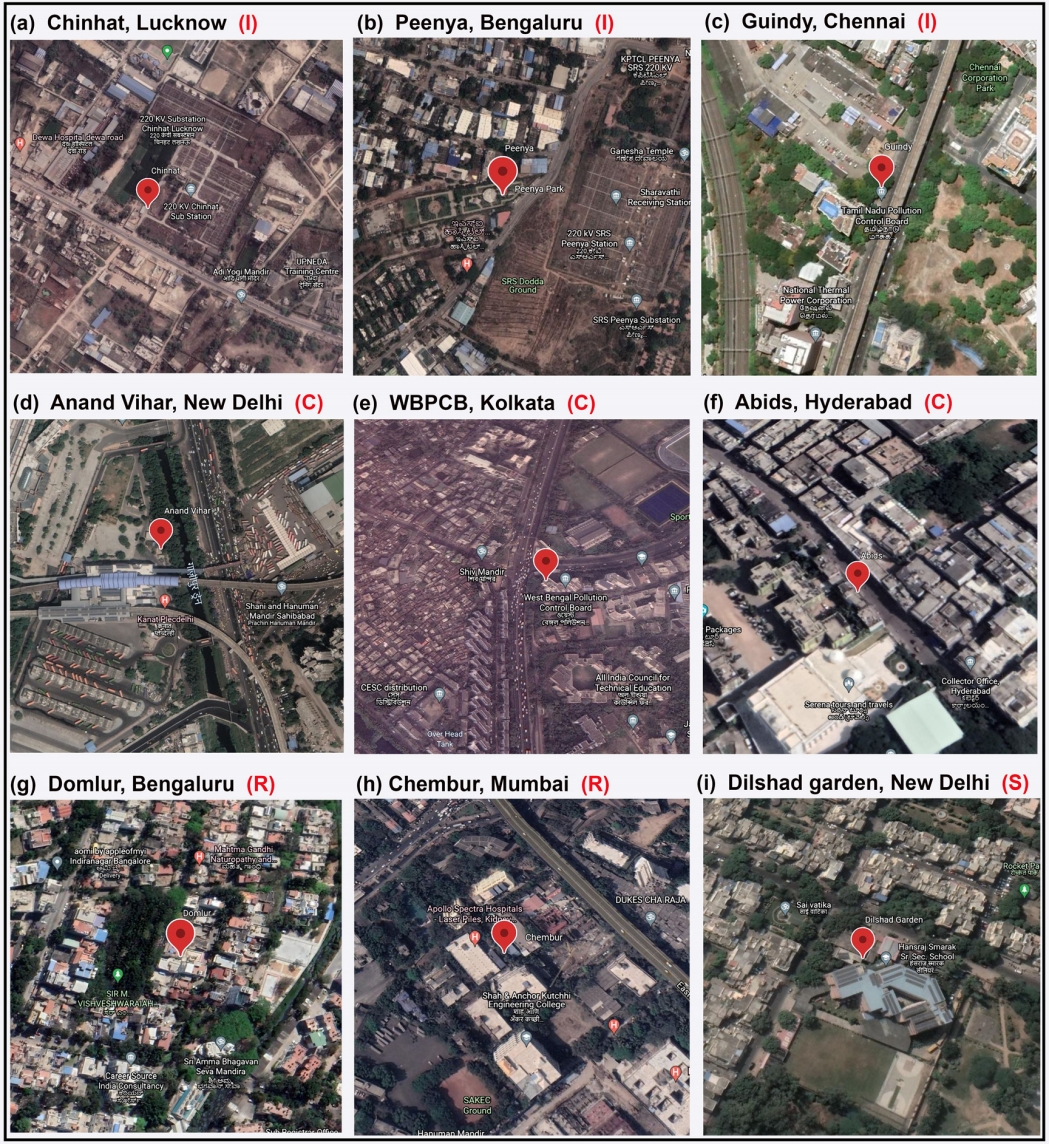
(Color online) Aerial photographs of the selected NANMN locations considered in this study. The approximate location of the noise terminals is shown by a red balloon.

Figures 1(a)–1(c) show the satellite (aerial) photographs of three representative noise terminal locations in I zone, namely, Chinhat (Lucknow), Peenya (Bengaluru), and Guindy (Chennai), respectively, while Figs. 1(d)–1(f) show the aerial photographs of three representative locations belonging to C zone given by Anand Vihar (New Delhi), WBPCB, Salt Lake City (Kolkata), and Abids (Hyderabad), respectively. The photographs in Fig. 1 were taken from Google Earth, and the red balloon symbol in these images denotes the approximate location of the noise terminals. Note that the noise terminals in Figs. 1(a)–1(f) are located in the vicinity of a major highway or near a traffic intersection point facing the road. Furthermore, all three I zone locations shown in Figs. 1(a)–1(c) have an electricity generating station, i.e., a power plant nearby which contributes to the ambient noise levels. Amongst the C zone locations, Anand Vihar is an important metro- and bus-station of New Delhi city, WBPCB is located right at a busy traffic intersection which connects different parts of Kolkata city, while Abids is one of the oldest business centers of Hyderabad city.

**FIG. 2.**
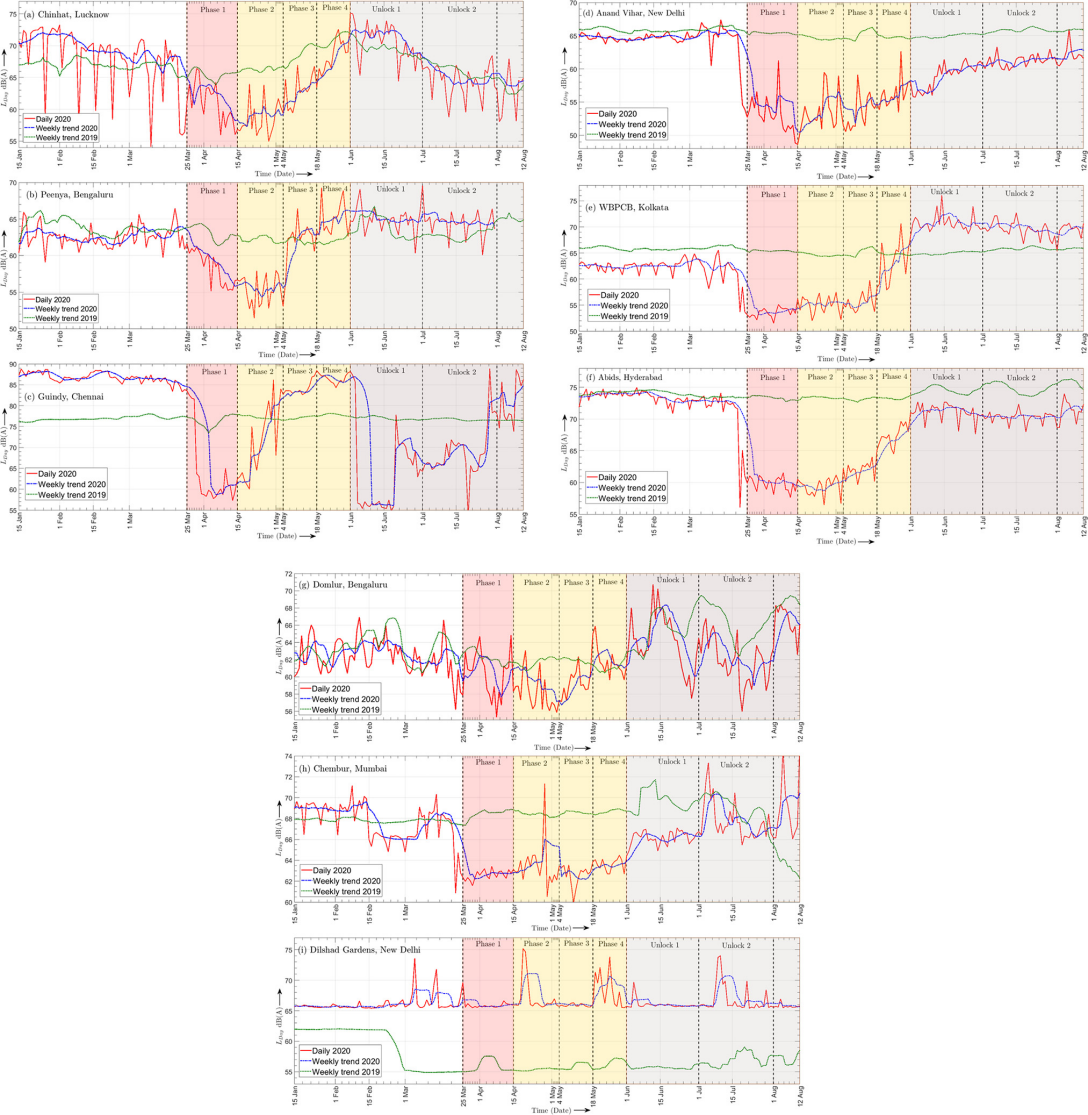
(Color online) Daily $L_{\mathrm{Day}}$ graphs for the year 2020 and the weekly trend graphs for the years 2020 and 2019 at the selected NANMN locations.

Figures 2(a)–2(f) show the daily day-time equivalent noise level $L_{\mathrm{Day}}$ graphs from 15 January 2020 to 12 August 2020 for the NANMN locations shown in Figs. 1(a)–1(f), respectively. Based on the day-time noise data, a short-term 7-day moving average was computed for the aforementioned period to smooth out the daily random fluctuations, and the weekly trend graphs are plotted alongside with the daily level graphs in Fig. 2 to more clearly bring out variation in levels due to the pandemic-induced lockdown. Figures 2(a)–2(f) also includes the weekly noise trend for the year 2019 during the same period, and a comparison of the trends for the years 2019 and 2020 during March-to-May highlights the specific role of the nationwide lockdown in reducing the ambient noise levels.

For both Chinhat and Peenya, it is observed from Figs. 2(a) and 2(b), respectively, that the noise levels decreased gradually during phase 1, and a minimum was observed sometime during phase 2 beyond which the levels began to increase almost linearly throughout phases 3 and 4 reaching steady daily fluctuations by the first week of June 2020, i.e., during the first month of UL when life was getting back to quasi-normal times—a similar trend was observed by Asensio *et al.*^6^ for different noise monitoring sites in Madrid. During the ULs, the mean noise levels were comparable to the pre-lockdown times. In contrast, for Guindy, Anand Vihar, WBPCB, and Abids locations, it is observed from Figs. 2(c)–2(f), respectively, that the levels dropped almost suddenly when phase 1 was declared, and minimum levels were reached sometime during this phase. For daily noise graphs pertaining to Guindy and Anand Vihar, it is observed that the levels began to increase from phase 2 onwards with the former location witnessing a rather steep increase, particularly during this phase while the latter experienced only a gradual increase until mid-June 2020. Guindy, however, again experienced a sudden fall in noise levels during ULs due to the containment measures adopted by Tamil Nadu state government as local increase in infections were reported. For Anand Vihar, however, the levels during ULs were comparable but marginally less than the pre-lockdown because the public transport services only partially resumed, people preferred working from home, and importantly, the local migrant labor population from other states were just beginning to return back. On the other hand, the graphs for WBPCB and Abids exhibited small daily fluctuations around the minimum and continued to stay low for a substantial part of phase 2. Following this, a rapid increase was observed during phases 3 and 4 until the levels stabilized during ULs, i.e., the graphs exhibited daily fluctuations around the mean levels. From the weekly trend 2020 graph in Fig. 2(e), it is evident that the mean was 5 dB(A) higher than the pre-lockdown level due to an increased local traffic-flux w.r.t. the pre-lockdown period, while in Fig. 2(f), it was highly comparable to the pre-lockdown because commercial activities resumed as before.

#### 1. Linear regression analysis

In order to quantify the approximate rate, i.e., dB(A)/day at which the noise levels fell during phase 1, and also to assess how quickly the levels recovered back when the restrictions began to be gradually eased from phase 2 onwards up to the end of lockdown, a piece-wise linear regression analysis was carried out to fit the daily day-time data for I and C zone locations. The regression graphs are not included for brevity, however, their slopes *m*, the duration (days) over which each linear-fit was obtained, and the correlation *R* with daily equivalent day-time data are listed in Table I. For the period starting just before the declaration of phase 1 up to the day at which the weekly trend graphs exhibited a minimum, a negative slope is obtained signifying a decreasing trend. In particular, Guindy exhibited the steepest decay rate given by −1.8 dB(A)/day while Peenya exhibited the gentlest decay rate given by −0.14 dB(A)/day. Similarly, for the period starting from the day when the weekly trends reached a minimum up to a certain part of the UL 1, a positive slope is obtained signifying an increasing trend. Here, again, Guindy exhibited the steepest recovery given by 0.55 dB(A)/day while for Anand Vihar, the levels increased at the slowest rate given by 0.11 dB(A)/day. A gradual ease of restrictions from phase 2 onwards implied that the economic activities resumed at a slow pace initially but gained momentum towards the end of the lockdown. This resulted in a day-by-day increase in traffic volumes which in turn caused the ambient levels to gradually increase, and a linear-fit is able to satisfactorily model the increasing noise trend as suggested from the corresponding correlation coefficients in Table I (7th column). Furthermore, NANMN locations across the country witnessed different rates at which the levels recovered because the pandemic management from phase 2 onwards was mainly carried out based on local government policy that varied for different states.

**TABLE I.** Slope *m* and the correlation coefficient *R* of linear regression fits for strict and relaxed lockdown phases for different NANMN locations.

Location, City and Zone	Strict lockdown phase			Relaxed lockdown phases		
	Duration	Slope m dB(A)/day	R	Duration	Slope m dB(A)/day	R
Chinhat, Lucknow (I)	22 Mar. to 19 Apr.	−0.26	0.5019	20 Apr. to 1 Jun.	0.29	0.8504
Peenya, Bengaluru (I)	24 Mar. to 25 Apr.	−0.14	0.5531	26 Apr. to 1 Jun.	0.24	0.6993
Guindy, Chennai (I)	23 Mar. to 9 Apr.	−1.8	0.8896	10 Apr. to 3 Jun.	0.55	0.8803
Anand Vihar, New Delhi (C)	22 Mar. to 15 Apr.	−0.23	0.5768	16 Apr. to 15 Jun.	0.11	0.6310
WBPCB-HQ, Kolkata (C)	19 Mar. to 1 Apr.	−0.91	0.8546	31 Mar. to 15 Jun.	0.26	0.8598
Abids, Hyderabad (C)	20 Mar. to 26 Apr.	−0.15	0.5226	27 Apr. to 18 Jun.	0.29	0.9411
Domlur, Bengaluru (R)	18 Mar. to 25 Apr.	−0.08	0.4470	26 Apr. to 18 Jun.	0.2	0.7634
Chembur, Mumbai (R)	10 Mar. to 1 Apr.	−0.57	0.8438	31 Mar. to 7 Jul.	0.06	0.6772

#### 2. Intraday noise level evolution

**FIG. 3.**
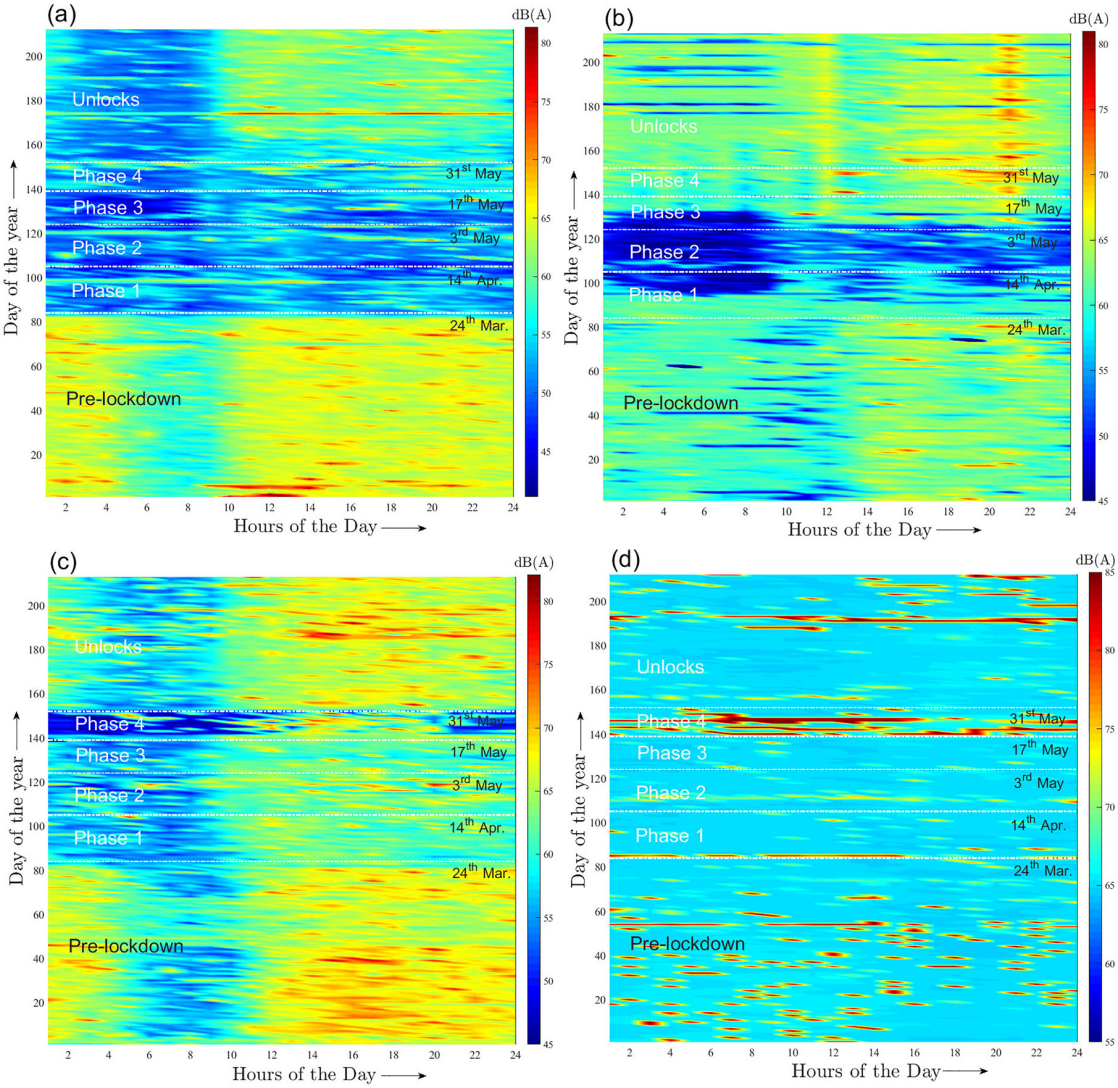
(Color online) $L_{\mathrm{Aeq},1\text{hr}}$ dB(A) intraday evolution at (a) Peenya, Bengaluru (I), (b) Anand Vihar, New Delhi (C), (c) Chembur, Mumbai (R), and (d) Dilshad Gardens, New Delhi (S). The horizontal axis corresponds to the hours of the day, vertical axis corresponds to the day in the year 2020 starting from beginning of January. The pre-lockdown period, phases of lockdown and ULs are indicated.

The lockdown considerably altered the intraday noise patterns at I and C zones, which is demonstrated by analyzing the hourly levels for one representative location belonging to each category. Figures 3(a) and 3(b) present the intraday noise level evolution for Peenya and Anand Vihar, respectively. Here, the *x* axis represents the hours of a day while *y* axis represents the days of the year beginning from 1 January 2020 up to the end of July 2020. The important dates which mark the declaration of different lockdown phases and start of UL is also indicated. Both parts (a) and (b) suggest that in the pre-lockdown period, part of the day from early morning 4 AM up to a little before noon was quiet while as expected, afternoon around 2 PM was the noisiest part due to maximum traffic, and a significant difference is observed between the quietest and noisiest parts. During the lockdown phases, however, the entire day was quiet where the levels were in general, significantly lower than the quietest part of the day during pre-lockdown. The occasionally noisy hours, especially in the afternoon during lockdown was due to plying of essential home delivery or emergency services. During ULs, the period from midnight to the 10 AM for Peenya was as quiet as the lockdown while the remaining part of the day was noisy but the levels were lower than the pre-lockdown. In contrast for Anand Vihar, the entire day witnessed roughly the same hourly levels comparable to the pre-lockdown values. Similar observations were also noted for other I and C locations analyzed in this work.

#### 3. Mean levels, maximum average noise reduction, and data variability

In order to compare the ambient noise scenario across different phases, the average noise level for a given period spanning several days is computed by taking the mean of the daily day-time and night-time levels. Table II shows the mean day-time levels ${\bar{L}}_{\mathrm{Day}}$ for the pre-lockdown period, individual phases 1 to 4, and ULs for different locations along with their respective standard deviations $s_{\mathrm{Day}}$, which is a measure of the amount of variation or dispersion from the mean levels due to the daily random fluctuations. With respect to the pre-lockdown, the maximum day-time average noise reduction occurred during phase 2 for both Chinhat and Peenya, and is given by 10.5 and 6.1 dB(A) respectively. For Guindy, Anand Vihar, WBPCB, and Abids, the maximum day-time average noise reduction occurred during phase 1, and is given by 11.6, 12.1, 8.8, and 13.8 dB(A), respectively. For all NANMN locations considered here except Chinhat, the daily night-time equivalent noise level $L_{\mathrm{Night}}$ graphs were found to exhibit the same trend or variation as their counterpart $L_{\mathrm{Day}}$ graphs where the correlation coefficient between the day- and night-time graphs was greater than at least 0.70. Although the night-time graphs are not shown here, we present the mean night-time levels ${\bar{L}}_{\mathrm{Night}}$ and the respective standard deviations $s_{\mathrm{Night}}$ for different periods in Table II. It is readily noted that Peenya, Guindy, Anand Vihar, WBPCB, and Abids reported a maximum night-time average reduction equal to 7 dB(A) during phase 2, and 11.2, 12.3, 6.8, and 14.1 dB(A) during phase 1, respectively.

**TABLE II.** Mean day-time ${\bar{L}}_{\mathrm{Day}}$ and night-time ${\bar{L}}_{\mathrm{Night}}$ indicators along with their respective standard deviations $s_{\mathrm{Day}}$ and $s_{\mathrm{Night}}$ for pre-lockdown period, lockdown phases 1 to 4, and ULs at different NANMN locations.

Location, city, and zone		Pre-lockdown	Phase 1	Phase 2	Phase 3	Phase 4	Unlocks
Chinhat, Lucknow (I)	D	69.7 ± 4.7	62.1 ± 3.1	59.2 ± 2.7	63.4 ± 2.6	69.8 ± 0.9	68.7 ± 4.7
	N	52.8 ± 2.8	51.6 ± 2.1	54.3 ± 1.2	55.0 ± 0.9	58.6 ± 1.6	60.4 ± 2.7
Peenya, Bengaluru (I)	D	62.0 ± 1.3	57.4 ± 2.9	55.9 ± 1.9	61.0 ± 3.1	63.6 ± 2.2	63.3 ± 1.8
	N	59.7 ± 1.8	57.6 ± 6.3	52.7 ± 3.1	55.9 ± 5.2	59.9 ± 0.4	61.6 ± 2.7
Guindy, Chennai (I)	D	86.9 ± 1.1	75.3 ± 14.6	77.5 ± 10.7	84.1 ± 1.4	86.7 ± 1.0	78.7 ± 14.0
	N	87.1 ± 1.0	75.9 ± 18.7	80.8 ± 10.0	85.6 ± 1.1	87.8 ± 1.6	81.6 ± 17.3
Anand Vihar, New Delhi (C)	D	66.5 ± 3.1	54.2 ± 2.8	54.4 ± 3.1	54.1 ± 2.7	59.1 ± 3.4	60.7 ± 1.9
	N	66.5 ± 3.1	54.2 ± 2.8	54.4 ± 3.1	54.1 ± 2.7	59.1 ± 3.4	60.7 ± 1.9
WBPCB-HQ, Kolkata (C)	D	62.4 ± 1.8	53.6 ± 0.8	55.6 ± 1.4	55.8 ± 1.7	66.9 ± 4.2	70.3 ± 1.6
	N	56.7 ± 1.1	49.9 ± 1.2	51.0 ± 1.4	51.4 ± 1.6	68.4 ± 3.0	70.1 ± 2.0
Abids, Hyderabad (C)	D	73.2 ± 2.6	59.9 ± 1.0	59.4 ± 1.6	62.0 ± 1.2	69.0 ± 0.7	70.9 ± 1.2
	N	66.0 ± 2.0	51.9 ± 1.2	52.9 ± 1.0	53.8 ± 0.8	56.9 ± 1.2	61.5 ± 2.0
Domlur, Bengaluru (R)	D	62.7 ± 2.0	61.0 ± 2.7	58.7 ± 2.1	59.3 ± 1.4	60.7 ± 0.7	64.6 ± 3.4
	N	59.7 ± 4.4	52.8 ± 2.8	51.3 ± 1.9	51.9 ± 2.1	51.9 ± 1.5	59.7 ± 4.4
Chembur, Mumbai (R)	D	68.0 ± 1.8	62.5 ± 0.4	65.4 ± 2.7	62.6 ± 1.0	63.8 ± 0.7	67.9 ± 2.1
	N	62.4 ± 1.0	58.2 ± 0.6	58.4 ± 1.1	58.5 ± 0.8	58.7 ± 0.4	64.0 ± 3.8
Dilshad Garden, New Delhi (S)	D	66.2 ± 1.2	66.1 ± 0.8	68.6 ± 3.2	65.8 ± 0.2	66.3 ± 1.2	66.8 ± 1.7
	N	66.3 ± 0.9	66.0 ± 0.8	68.7 ± 3.4	65.6 ± 0.1	65.5 ± 0.1	66.9 ± 2.1

The coefficient of variation (CV) given by $s_{\mathrm{Day}}/{\bar{L}}_{\mathrm{Day}}$ or $s_{\mathrm{Night}}/{\bar{L}}_{\mathrm{Night}}$ was also computed for pre-lockdown period, phases 1 to 4 and ULs. In Table II, the period exhibiting the largest CV for a given location and day- and night-time interval is underlined which is usually the ULs, phase 4 or pre-lockdown due to a greater daily fluctuations. In general, the stringent phase 1 has a smaller CV because the daily fluctuations were relatively low.

### B. Residential and silence zones

Figures 1(g) and 1(h) show the aerial photographs of two representative noise terminal locations in well-developed R zones, namely, Domlur (Bengaluru) and Chembur (Mumbai), respectively, while Fig. 1(i) shows the photograph of a location in Dilshad Gardens (New Delhi), a S zone. Note that by a S zone, we refer to a region of at least 100 m radius measured from the nearby educational institutions, hospitals or law courts. For the R and S zones presented in Figs. 1(g) and 1(i), respectively, the noise terminals are located somewhat towards the interiors and surrounded by buildings, considerably away from the main road whilst for the R zone shown in Fig. 1(h), the noise terminals is somewhat closer to a main road.

Figures 2(g)–2(i) show the daily $L_{\mathrm{Day}}$ graphs superposed with the weekly trend for the same duration 15 January to 12 August 2020 for the locations shown in Figs. 1(g)–1(i), respectively. Figure 2(g) seems to suggest that the lockdown produced only a marginal noise reduction which is not surprising given the noise terminal location. Here, the levels continued to decrease gently until a minimum was reached towards the end of phase 2 followed by a gradual increase to mid-June 2020, refer to the slopes for this location presented in Table I. Furthermore, it is noted from Table II that the maximum day- and night-time average noise reduction occurred during phase 2, and is given by 4 and 8.4 dB(A), respectively, while the ULs have the largest CV. In contrast, Chembur was characterized by a sudden decrease in levels at the onset of phase 1 as shown in Fig. 2(f) and a noticeable reduction was observed throughout the lockdown. However, phase 2 witnessed increase in daily fluctuations causing this phase to exhibit the largest CV. The levels started climbing back with a very gentle slope from the beginning of phase 2 onwards and reached steady daily fluctuations only towards the end of UL 1. The lockdown, however, did not alter the intraday noise pattern at Chembur although during different phases, noisy parts of the day were much quieter than the pre-lockdown times and ULs as may be observed from Fig. 3(c). The maximum day- and night-time average reduction for this R zone was observed during phase 1, and is given by 5.5 and 4 dB(A), respectively. Finally, a comparison of the 2019 and 2020 weekly trends during March to May in Figs. 2(g) and 2(h) further demonstrates the effect of lockdown on ambient noise reduction at the R zones.

A nearly flat $L_{\mathrm{Day}}$ graph shown in Fig. 2(i) suggests that the acoustic environment at Dilshad Gardens was not impacted at all by the lockdown, notwithstanding the presence of occasional peaks which is most likely attributed to plying of emergency medical services on certain days. This conclusion can also be arrived at by observing the intraday noise evolution pattern in Fig. 3(d) as well as from the average noise levels and standard deviation values reported in Table II. Note that the phase 2 exhibits the largest CV. However, the 2019 weekly trend graph was significantly lower than its 2020 counterpart throughout the study period—an increase in ambient levels during 2020 is attributed to local infrastructural development.

### C. Phase-wise boxplot comparison

**FIG. 4.**
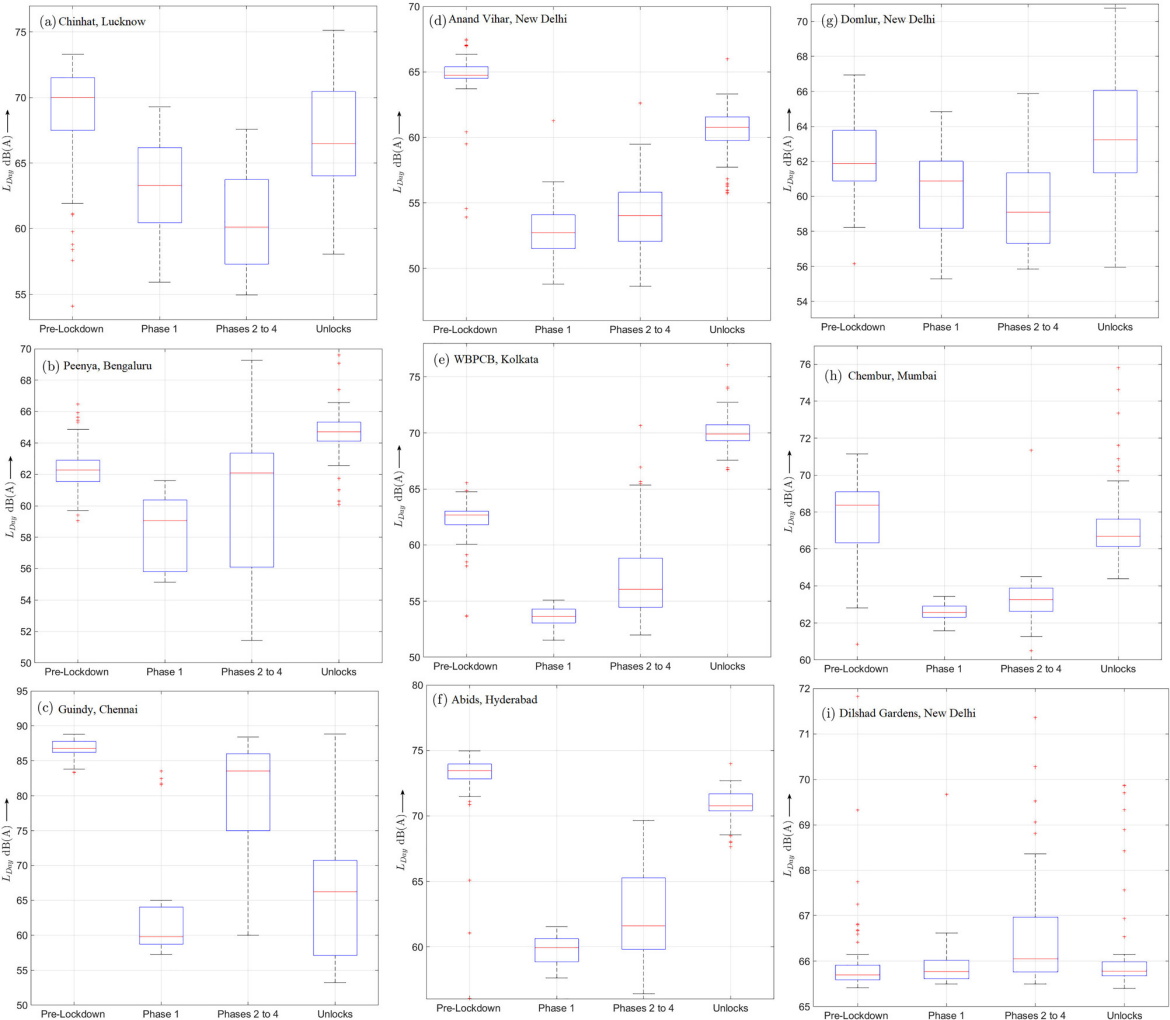
(Color online) Boxplot values of $L_{\mathrm{Day}}$ for pre-lockdown period, phase 1, phases 2 to 4, and ULs at different NANMN locations.

Figures 4(a)–4(i) present a comparison between the boxplots of the day-time levels $L_{\mathrm{Day}}$ during the pre-lockdown, phase 1, combined phases 2 to 4 and the ULs for the NANMN locations shown in Figs. 1(a)–1(i), respectively. It is readily observed from Fig. 4 that the median noise levels (shown by red lines) exhibit a similar variation trend from the pre-lockdown period up to the ULs as their counterpart weekly trend graph for the year 2020 shown in Fig. 2. Furthermore, during certain periods, the boxplots display skew as may be observed from the location of first and third quartiles (shown by blue lines) w.r.t. the median. An important feature of the boxplots shown in Fig. 4, however, is the presence of outliers, i.e., extreme values which mainly occur during the pre-lockdown and/or the ULs due to a greater daily fluctuations. For instance, in the case of Dilshad Gardens analyzed in Fig. 4(i), several outliers located above the top whisker were observed except during the strict phase 1 which is consistent with the presence of occasional peaks in Fig. 2(i).

### D. Summarized results for other NANMN locations

The $L_{\mathrm{Day}}$ and $L_{\mathrm{Night}}$ graphs for the remaining 61 NANMN locations belonging to I, C, R, and S zones were also analyzed although their results are not included. It was found that the graphs exhibit the same trend as those presented in Figs. 2(a)–2(h) with other S zones experiencing a marginal noise reduction during the lockdown phases. However, there were some locations where it was observed that the lockdown did not have a significant impact on the acoustic environment; for such exception cases, the graphs exhibited almost the same daily trend during lockdown phases as observed during the pre-lockdown. Furthermore, for a few NANMN locations which includes a couple of S zones having an important public hospital nearby, the levels significantly increased during the later phases of the lockdown which is most likely attributed to an increased local traffic flux due to incoming/outgoing patients who required hospitalization arising from COVID-19 symptoms.

## V. ENVIRONMENTAL NOISE PERCEPTION SURVEY

This section reports the results of an online cross-sectional survey named *COVID19 Noise Perception Survey* conducted with a view to gain a qualitative insight on the effect of lockdown on the environmental noise levels across Indian cities, the dominant outdoor noise sources before and during the lockdown, and the impact on health and well-being due to the changes in noise levels during the lockdown. To this end, a short questionnaire was created using a Google form which was send to potential participants pan-India through e-mails, social media posts, and messenger services during the lockdown until the desired sample size n=1068 was reached which corresponds to a 95% confidence level within a confidence interval, i.e., a margin of error equal to ± 3%, see Ref. 31. In this national survey, only healthy adults, i.e., people with age 18 and above with no prior hearing impairments were requested to participate. Furthermore, 75.7% participants were between 21 to 40 years, 12.7% were between 40 to 60 years, and 5.8% each belonged to the age-groups 20 or less or 60 and beyond. Note that there was an unintentional gender bias; 73.7% participants were male and 25% were female, while the remaining preferred not to disclose their gender.

Part (i) of SuppPub1.jpg^27^ depicts the distribution of participants across India wherein it is readily observed that all important cities and small towns were covered. Furthermore, 81.7% participants identified themselves to be living within the city which includes residential (newly developed) areas, old or commercial areas as well as near shopping malls, cinemas and hospitals. The remaining 18.3% participants lived in the city outskirts, i.e., suburban areas and industrial regions.

### A. Survey responses

**FIG. 5.**
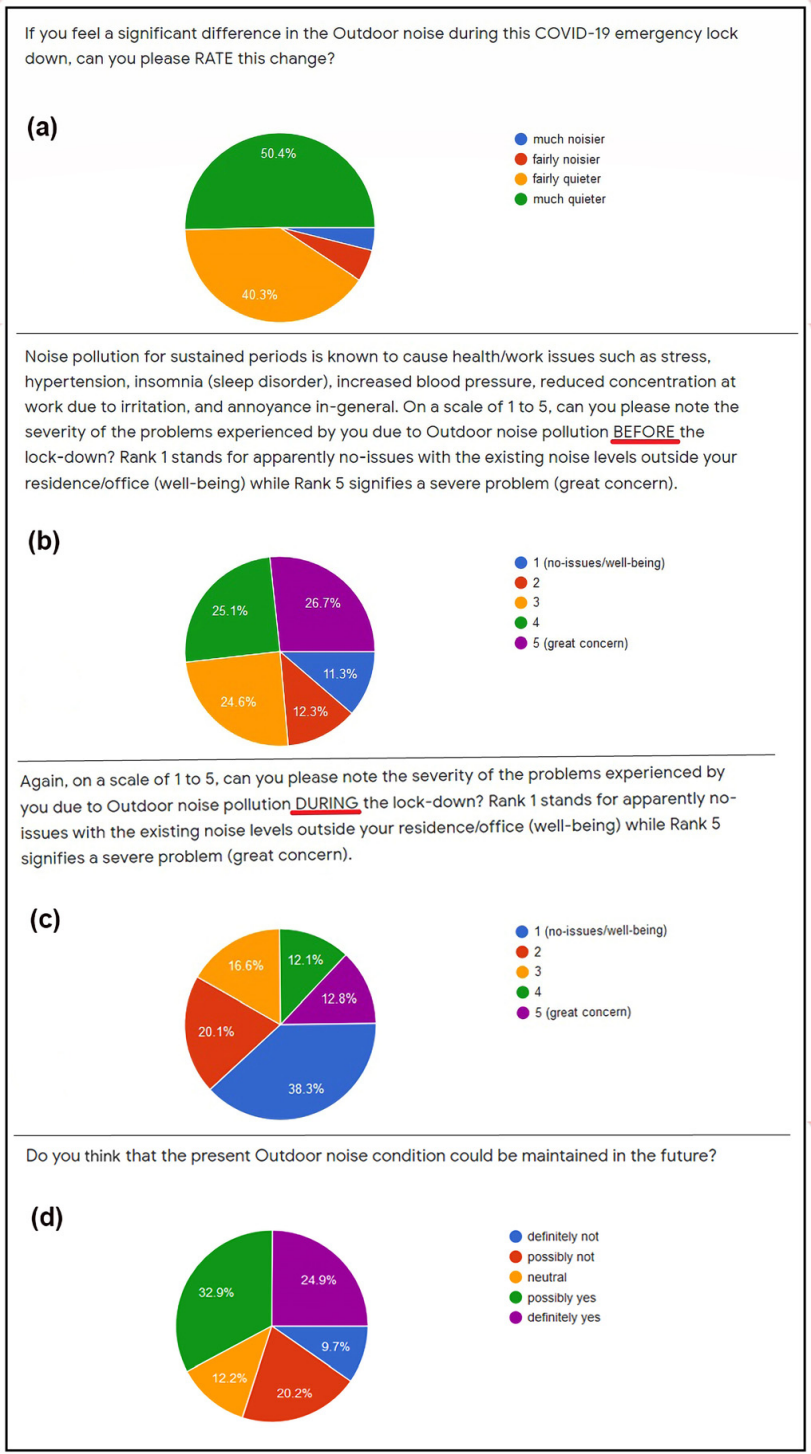
(Color online) Pie graphs showing the responses to (a) Q.1, (b) Q.2, (c) Q.3, and (d) Q.4.

The survey primarily comprised of four easy-to-answer questions which along with their respective response pie charts are shown in a consolidated manner in Fig. 5. The pie chart in Fig. 5(a) reveals that 50.5% participants perceived the lockdown to be *much quieter* while 40.2% participants perceived it to be *fairly quieter*, implying that at least 90.7% of the sample population felt that the outdoor or environmental noise reduced due to the lockdown restrictions. Indeed, this response correlates quite well with the results obtained from the NANMN data presented in Figs. 2 and 3 which indicate a significant reduction in ambient noise levels at all I, C and R zones considered in this work during lockdown. Further recall from Sec. IV that the maximum average day- and night-time noise reduction varied widely with some locations witnessing only 4 dB(A) decrease while other locations experienced a reduction in excess of 10 dB(A). Additionally, from Fig. 2 it can be seen that on certain days either during phase 1 or 2, Guindy, Anand Vihar, and Abids witnessed a reduction up to 31, 15, and 18 dB(A), respectively, w.r.t. the mean levels during pre-lockdown while the graphs for Domlur and Chembur suggests a much smaller reduction—these observations further corroborate the aforementioned responses. On the other hand, less than 10% felt that the lockdown was either *fairly noisier* or *much noisier* which is also supported by the NANMN data because for a few locations there was a significant increase in levels due to the reasons noted earlier.

The pie chart responses shown in Figs. 5(b) and 5(c) together highlight the indirect benefit that the lockdown had on the public-health and well-being. From Fig. 5(b) it is readily seen that nearly 52% of the sample population had a high-to-great noise annoyance issues during the pre-lockdown times which is not surprising because Indian cities and even smaller towns are noisy where the ambient noise levels usually exceed, or are very close to the maximum permissible limit as observed from $L_{\mathrm{Day}}$ graphs for most locations in Fig. 2. Only 11.4% participants did not experience any noise-related health issues. During lockdown when the outdoor environment was quieter in general, the proportion of people who did not experience any health-issues or annoyance arising out of ambient noise levels jumped significantly to 38.4%, i.e., 3.37-fold increase. This response is also supported by the NANMN results in Fig. 2 which shows that for all I and C zones, the lockdown bought down the ambient noise levels to be much lower than the prescribed maximum day-time limit which is directly related to a reduced noise-induced annoyance. Note that 25% participants still expressed concerns due to the ambient noise as observed from Fig. 5(c). However, the environmental noise-related issues were not the only concern during lockdown because a significant percentage of the population may also have faced anxieties and higher-stress levels while being confined to their homes in those highly uncertain times which indirectly affected people and their perception of the environment.^22^ In fact, for people living near busy areas who were accustomed to a constant exposure to roadside noise were suddenly forced to live in a completely different acoustic environment which was much quieter, this may well have also caused mental-health issues arising out of feelings of confusion or loneliness.

The participants were also asked to rank the different outdoor noise sources before and during the lockdown. As anticipated, the response (not shown here) revealed that during the pre-lockdown, the traffic noise was perceived as the dominant contributor followed by shops and commercial operations. However, during the lockdown phases, the responses suggested that vehicles, commuters and commercial operations contributed least to the ambient levels while the noise produced by animals and bird chirping became noticeable. Indeed, this response is consistent with the traffic data for metropolitan cities like New Delhi, Mumbai, Pune, and Bengaluru provided by TomTom, a multinational developer of maps and location technology which revealed that in the year 2020, April and May were the least congested months while January was the most congested month. Note that the average traffic congestion across these four cities during January, April and May were given by 64%, 3%, and 15.5%, respectively.^32^ Now since people were working from their homes^33^ and managing with only essential home deliveries, schools and universities running online, the survey also aimed to know the opinion of participants whether the resultant low outdoor noise levels during the lockdown can also be maintained in the near-future. The response pie chart in Fig. 5(d) revealed that only 32.4% participants felt it was difficult to maintain similar noise levels as those observed during lockdown in regular times, i.e., when the COVID-19 crisis is over, while nearly 58% participants opined that such an arrangement could well be feasible. As it turned out, the NANMN results in Fig. 2 support the views of the minority proportion because the levels during ULs were comparable to pre-lockdown for all locations considered.

## VI. CONCLUSIONS

To summarize, this study has analyzed the impact of a little over two-month nationwide lockdown in India from 25 March to 31 May 2020 on the urban noise levels at selected representative locations across seven major Indian cities based on the data recorded by the National Ambient Noise Monitoring Network (NANMN). An analysis of the daily day-time noise graphs indicated that all commercial (C), industrial (I), and residential (R) zones considered here experienced a reduction in the ambient levels during the lockdown phases, which were much quieter than the corresponding period in the year 2019. Depending upon the noise terminal location, the maximum average day-time reduction widely ranged from 4 to 13.8 dB(A) while the counterpart values for night-time varied from 4 to 14.1 dB(A) w.r.t. the pre-lockdown mean levels. When the lockdown restrictions were somewhat relaxed from phase 2 onwards, the noise levels at I, C, and R locations increased almost linearly, reaching steady daily fluctuations during unlocks (ULs) exhibiting a trend similar to the pre-lockdown. Furthermore, depending upon the then prevailing local conditions and behavioral pattern, the mean noise levels during ULs were either marginally lower, comparable, or noticeably higher than the pre-lockdown period. The silence (S) zone considered here was not impacted throughout the lockdown. The conclusions drawn from the NANMN data are in agreement with the subjective results, i.e., responses to an online survey, which suggested that 90% participants perceived the lockdown to be quieter due to limited traffic and imposition of overnight curfews.

Although ambient noise measurements at many more locations across the country would deliver greater insights on the modified acoustic environment, it can undoubtedly be concluded that during the lifetime of the lockdown, the local soundscape of normally loud commercial or industrial areas and a few noisy residential regions across Indian cities were considerably altered, thereby bringing down the noise pollution levels. It is then desirable that a careful planning for the post-COVID scenario be carried out which includes traffic management, preferably at a local government level to ensure that in the long-term, considerably lower ambient noise levels are consistently observed resulting in far less annoyance issues arising out of environmental noise pollution when the situation becomes completely normal.
